# Spontaneous first trimester miscarriage rates per woman among parous women with 1 or more pregnancies of 24 weeks or more

**DOI:** 10.1186/s12884-017-1620-1

**Published:** 2017-12-22

**Authors:** Judy Slome Cohain, Rina E. Buxbaum, David Mankuta

**Affiliations:** 1 Alon Shvut 37, Israel; 2 Shaare Zedek Medical Center, Jerusalem, Israel; 3 Dept Obstetrics and Gynecology, Hadassah Medical Center, Hebrew University, Jerusalem, Israel

**Keywords:** First trimester spontaneous miscarriage, Grand multiparity, Infertility, Maternal age, Non recurrent miscarriage

## Abstract

**Background:**

The purpose of this study was to quantify spontaneous first trimester miscarriage rates per woman among parous women. A vast amount of data has accumulated regarding miscarriage rates per recognized pregnancy as well as about recurrent miscarriage. This is the second study of miscarriage rates per woman in a parous population and the first study of recurrent and non-recurrent, spontaneous first trimester miscarriage rates per woman in a large parous population.

**Methods:**

Extraction of the following variables from all delivery room admissions from both Hadassah Medical Centers in Jerusalem Israel, 2004–2014: # of first trimester spontaneous miscarriages, # live births; # living children; age on admission, pre-pregnancy height and weight, any smoking this pregnancy, any alcohol or drug abuse this pregnancy, blood type, history of ectopic pregnancy, history of cesarean surgery (CS) and use of any fertility treatment(s).

**Results:**

Among 53,479 different women admitted to labor and delivery ward, 43% of women reported having had 1 or more first trimester spontaneous miscarriages; 27% reported having had one, 10% two, 4% three, 1.3% four, 0.6% five and 0.05% reported having 6–16 spontaneous first trimester miscarriages. 18.5% had one or more first trimester miscarriages before their first live birth. Eighty-one percent of women with 11 or more living children experienced one or more first trimester miscarriages. First trimester miscarriage rates rose with increasing age, increasing parity, after previous ectopic pregnancy, after previous cesarean surgery, with any smoking during pregnancy and pre-pregnancy BMI ≥30.

**Conclusions:**

Miscarriages are common among parous women; 43% of parous women report having experienced one or more first trimester spontaneous miscarriages, rising to 81% among women with 11 or more living children. One in every 17 parous women have three or more miscarriages. Depending on her health, nutrition and lifestyle choices, even a 39 year old parous woman with a history of 3 or more miscarriages has a good chance of carrying a future pregnancy to term but she should act expediently.

## Background

A search on Pubmed.com for the search words ‘recurrent miscarriage’ provides 8000 citations regarding the 1% of women who suffer recurrent miscarriage, the phenomenon of 3 or more consecutive first trimester miscarriages [1]. A second search on Pubmed.com for the term ‘Miscarriage Rates’ reveals a large number of studies on miscarriage rates per pregnancy, establishing that 15% of pregnancies typically recognized after the woman misses her period, either by ultrasound or urine pregnancy test, spontaneously miscarry in the first trimester. The spontaneous miscarriage rate varies between from 10% to 20% where 10% refers to late recognition of pregnancy and 20% refers to research involving routinely testing for pregnancy before 4 weeks or 4 weeks after the last menstrual period [1, 2].

Computerized patient records, tested for reliability, have been in use at Hadassah Hospital Delivery Rooms since 2004, where about 5000 women deliver per year. The labor and delivery units at Hadassah in Jerusalem admit women of 24 weeks of pregnancy or more. The patient record at Hadassah has separate fields for first trimester spontaneous miscarriage, induced abortion, second trimester spontaneous miscarriage and stillbirth over 500 g. Therefore, this database is equipped to carry out the goal of this study, which was to identify the prevalence of spontaneous first trimester miscarriage per woman in parous women.

## Methods

A search for miscarriage rates per woman was undertaken on Pubmed using the search words: Miscarriage, Recurrent Miscarriage, Non-recurrent miscarriage, and First Trimester Miscarriage.

A retrospective analysis of all admissions to the delivery room 2004–2014 in 2 Hadassah hospitals, 4 miles apart, sharing the same name, administrators, similar populations and the same electronic medical record. The patient record is filled in by the Certified Nurse Midwife who admits the client. The Certified Nurse Midwife asks and then documents the client’s answers in the computer file. The information entered in the patient file is confirmed during the doctor’s admission interview of the patient. Double questioning of the client by both the midwife and the admitting doctor is routine, and takes place almost without exception, as a method of ensuring the reliability of the data. On subsequent admissions of the same patient, the patient file is reopened and the patient history is updated by the admitting doctors and midwives. For women who delivered more than once at a Hadassah hospital during the study period, the most recent admission was included and the older file was excluded from the study. Subsequently, patient records were anonymized and de-identified prior to the computer extraction of the variables from the computerized charts onto an Excel spreadsheet.

The population was previously described [3]. In brief, the population benefits from socialized prenatal care equally available to all citizens, and low rates of teenage pregnancy, single mothers, alcohol abuse, drug abuse, and sexually transmitted diseases.

Variables extracted from each chart: identity number, # of first trimester spontaneous miscarriages, # live births; # living children; age on admission, pre-pregnancy height and weight, any smoking, any alcohol or drug abuse, blood type, history of ectopic pregnancy, history of cesarean surgery and use of any fertility treatment(s). Miscarriages which took place before the first live birth were available and extracted. After first births, the database did not distinguish the temporal order of miscarriage e.g. which birth the miscarriage preceded. The women were not asked whether they confirmed the pregnancies that resulted in recognized miscarriage by ultrasound or urine blood test or both.

The doctor’s interview with the patient was the first reliability check of the data. The second check was performed by the authors comparing the actual files to the extracted data for 114 randomly selected patient files. The computer expert who maintains the Hadassah Medical Center’s server and archives and Judy Slome Cohain compared the charts of the women with the highest and most unusual rates of miscarriage starting from those who had experienced 17 (*n* = 1) 16 (*n* = 4) 15 (*n* = 8) 14 (*n* = 15), 13 (*n* = 56) and 12 (*n* = 87) miscarriages. We stopped after comparing the data in 114 patient records to that in the Excel spreadsheets due to time limitations. In every case Excel extraction reflected the data in the actual charts, so we concluded that we could rely on the extracted data.

There was no known conflict of interest or bias by the authors for any particularly outcome. No known cultural taboos exist in Israel that would compel women to either under or over report spontaneous miscarriages. At Hadassah, the data is entered by motivated midwives and doctors interested in promoting the use of the patient records for research. Formal ethical approval for the use of the database for the purpose of this research was received by the Hadassah Medical Organization Helsinki Committee Institutional Review Board on April 14, 2014.

The variables were extracted using Excel tables. The relative risk, RR, of the possible risk factors was calculated using the standard equation.

## Results

There were 65,541 admissions during the study period. Three hundred fourteen were excluded because they were women who were admitted more than once during the study period, leaving 65,227 women. There were a total of 237,755 documented pregnancies among 65,227 different women, documenting an average of 3.65 pregnancies per woman admitted, including 2.07 living children per woman before the study admission for delivery. Eighteen percent of the 65,541 patient files had a blank field for ‘spontaneous miscarriage’ meaning either no miscarriages or the field was accidently left empty. Calculations of miscarriage rates were calculated among the 53,479 women for whom a value was entered in the field for ‘spontaneous miscarriage’. There were 35,862 miscarriages reported among the 237,755 pregnancies, finding that 15% of the documented pregnancies ended in first trimester miscarriages per pregnancy.

### Demographics

The average age on admission was 30.4 years, range 14–53. The percent of women with a history of 1 or more previous cesareans was 11%. There were 4.5% smokers, 0.17% drug abusers, 0.2% alcohol abusers, 0.9% of women with a history of 1 or more ectopic pregnancies, 7% of women underwent some fertility treatment, documented as either Clomid, Pergonal, IVF, egg donation, sperm donation, or other. Average pre-pregnancy BMI was 23.9. The average height was 1.66 m. Ten percent were Rh negative.

**Fig. 1:**
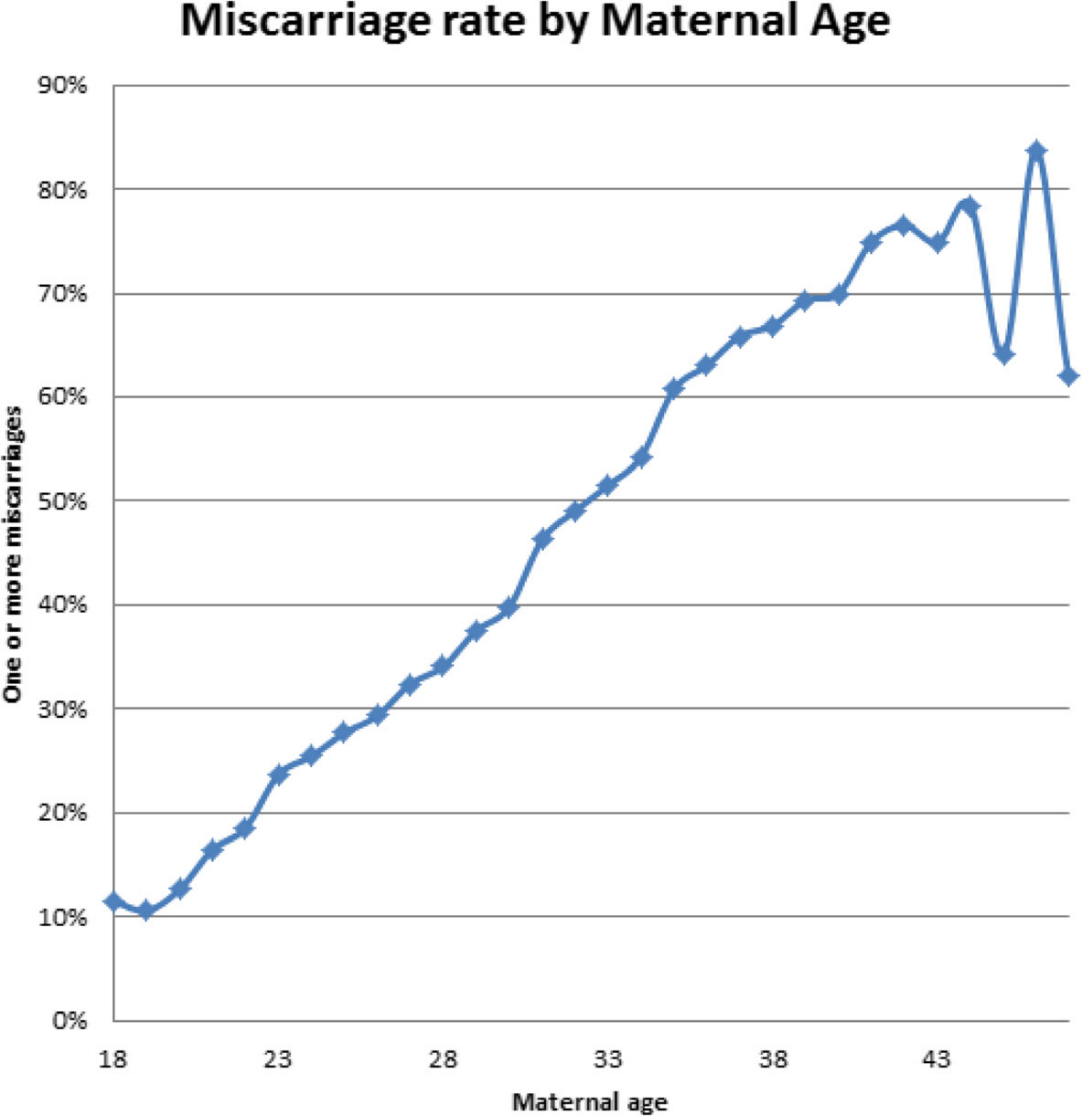
Documents the relationship between women who experienced one or more miscarriage by age 18–46

**Fig. 2:**
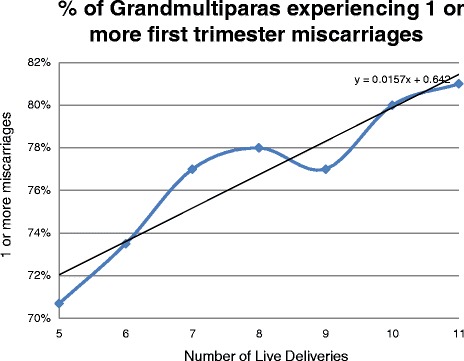
Shows the relationship between number of first trimester miscarriages and number of live births among grandmultiparous women

Miscarriage rates directly increased with age and with parity as can be seen in Table 1, Figs. 1 and 2. Figure 1 documents the relationship between women who experienced one or more miscarriages by age 18–46. Figure 2 reports the relationship between number of first trimester miscarriages and number of live births among grand multiparous women. Higher rates of miscarriages per woman were found in this study compared to the 2 previous studies on this subject as seen in Table 2. As is seen in Table 3, women who had 1–5 miscarriages had an average of 3 children, those who experienced 6–16 miscarriages, had an average of 4 children. A slight but statistically increased incidence of miscarriage was associated with:

- Previous CS (RR 1.66, 95% Cl 1.63–1.70, *P* < 0.0001),
- History of 1 or more ectopic pregnancy (RR 1.62, 95% CI 1.55–1.70, P < 0.0001),
- Any smoking this pregnancy (RR 1.27, 95% Cl 1.15–1.40, P < 0.0001), and
- Pre-pregnancy BMI ≥30 (RR 1.23, 95% Cl 1.09–1.38, *P* = 0.0006).
- Miscarriage rates were not increased among women undergoing 1 or more fertility treatment (RR 0.93, 95% CI 0.90–0.97, *P* = 0.0019) or for any blood type. All blood types, O+, A+, B+, AB+, O-, A-, B-, AB- experienced the same incidence of 1 or more miscarriages.

**Table 1:** 0 to 5 Miscarriage Rates by Parity 0 to 11

Parity=	N=	Miscarriages = 0	=1	=2	=3	=4	=5
0	14,881	81.5%	14%	3.4%	1%	0.3%	0.1%
1	12,392	63%	26%	7%	2.4%	0.7%	0.4%
2	10,515	49%	34%	11.6%	3.6%	1.4%	0.5%
3	6579	40%	38%	14%	5%	1.7%	0.7%
4	3880	33.7%	39%	17%	6.4%	2.8%	1%
5 to 11	5232	26%	38.5%	20%	10%	3.9%	1.7%
Total	53,479	57%	27%	10%	4%	1%	0.4%

**Table 2:** Miscarriage rates per woman in our study compared to the 2 previous studies

	N =	Misc = 0	=1	=2	=3	=4	5
Blohm 2008 Sweden	320	75%	19%	4%	1%	0.3%	0
Gunnarsdottir 2014 Sweden Primparous only	619,587	87%	11%	2%	≥3 = 0.6%		
Hadassah 2015 Israel	53,479	57%	27%	10%	4%	1%	0.4%

**Table 3:** 6–16 miscarriages / woman vs. Living children and Fertility Treatments

Miscarriages	N=	Avg # Living Children	# fertility tmt(s)
6	144	4.2	2/144
7	67	1.1	7/67
8	32	4.7	1/32
9	16	3.6	2/16
10	11	3.1	1/11
12	1	2	0
13	3	4.6	0
14	4	5.5	0
15	1	6	0
16	1	5	0

## Discussion

In this population, 43% of women reported a history of 1 or more recognized spontaneous first trimester miscarriages. If underreporting occurred, perhaps due to denial, forgetfulness, and/or miscarriage mistaken for delayed menstruation, the number of parous women reporting spontaneous first trimester miscarriage rate might approximate 50%. The findings show miscarriage to be widespread. This concurs with current research showing about 50% [2] to 60% [4] of miscarriages are the result of random fetal chromosomal abnormalities incompatible with life.

Advanced maternal age has been documented to be the strongest risk factor for miscarriage, with women over 42 years experiencing 50% miscarriage rates and women aged 45 associated with 75% rates [5, 6]. This was confirmed by our data.

The number of miscarriages per woman increases with parity. Women of higher parity are older than women of lower parity in general, which may explain this association.

Recurrent miscarriage is defined as three or more consecutive miscarriages and is consistently previously reported as affecting 1% of women and was thought to be predictive of high rates of future miscarriage [1]. In contrast to this, 6% of women in this population had three **or more** recurrent or non-recurrent miscarriages and went on to deliver at least one more child. Regan et al.’s extensive body of work on recurrent miscarriage states: “The most predictive factor for spontaneous abortion is a previous spontaneous abortion. Since the most important predictive factor for spontaneous abortion is a previous spontaneous abortion, the outcome of a woman’s first pregnancy **has profound consequences** for all subsequent pregnancies” [7]. Regan’s work studies populations of more limited generalizability and ignores maternal age, the most predictive factor for first trimester miscarriage [5, 6].

The largest study of recurrent miscarriage concluded that 16% of women will miscarry the next pregnancy after their first miscarriage, 25% of women are expected to have a third consecutive miscarriage after 2 miscarriages, and 45% are expected to have a fourth and 54% are expected to have a fifth [8]. The paper admits that 17% of the repeat miscarriages were not repeat miscarriages but re-admissions for diagnostic follow-up testing [8]. Even if their rates were adjusted to accurately reflect the correct numbers as well as ages of the women, this research is only about recurrent miscarriage rates in the Danish population where miscarriage is routinely treated with dilation and curettage. Dilation and curettage could theoretically increase subsequent recurrent miscarriage due to the 25% rate of adhesions left after a single dilation and curettage [9], the same as after a single cesarean [10]. To date, the influence of the routine use of dilation and suction vacuum aspiration on subsequent spontaneous miscarriage is unknown [11, 12].

The bulk of published research on recurrent miscarriage concludes that recurrent miscarriage is associated with high rates of subsequent recurrent miscarriage and ‘profound consequences’ is a possible euphemism for inability to produce a live child. Quite the opposite was found in this study. Recurrent and non-recurrent miscarriage were associated with high parity. Eighty-one percent of women with 11 or more living children experienced one or more first trimester miscarriages. About 7% of grand multiparous women with 5 or more children had 3 or more miscarriages and 2% experienced as many as 5 miscarriages.

The 2 previous studies exploring the rate at which women experienced one or more non recurrent miscarriages are from Sweden [13, 14]. One is a prospective study intending to study 474 **non-randomly** recruited ‘volunteers’ to age 29, of unknown generalizability, and 320 **non-randomly** recruited volunteers up to age 39. Nearly half (48%) of the 794 women were lost to follow up during the study [13]. The second study is a large study group but only concerned with primiparous women [14]. The miscarriage rates in both studies might be artificially lowered by Sweden’s induced abortion rate of 20%, since in the absence of induced abortion, spontaneous miscarriage rates are higher.

This study found higher miscarriages rates per woman than the 2 previous studies on this topic. The 2 previous studies were performed in Sweden where the fertility rate is 1.8 compared to the Israeli rate of 2.96 for 2009 and 3.04 for 2012. The lower miscarriage rates in Sweden may be partially or fully explained by their lower fertility rates and higher induced abortion rates. Sweden has an induced abortion rate which is double the rate in Israel: 20% vs. 10% (http://www.johnstonsarchive.net/policy/abortion/ab-israel.html). Induced abortion is frequently performed before spontaneous miscarriage occurs, lowering miscarriage rates. One study suggests that as many as 25% of induced abortions would have spontaneously miscarried had an induced abortion not been performed [15]. The limitations of the Swedish studies, were overcome by the use of our large sample of every parity in a reliable database with a separate field for both induced and spontaneous miscarriage.

We looked at possible factors affecting the risk of spontaneous miscarriages in our population: Our data documented a small but significant increase in miscarriage among women who had a history of a previous cesarean. A 2013 review [13] on the subject found insufficient evidence to determine if CS increased the risk of subsequent miscarriage although the risk of miscarriage was increased following CS in the multinomial logistic regression analysis [16]. A possible mechanism to explain increased miscarriage after previous CS could be uterine scarring, the mechanism that was used to explain the doubling of the rate of third trimester unexplained stillbirth after previous cesarean [17].

As would be expected, women with a history of ectopic pregnancy, smoking and BMI ≥30 experienced significantly more first trimester miscarriages.

The rates of miscarriage among women having fertility treatment were similar to the rates for women not having any fertility treatments. Four thousand four hundred sixty-six (7%) underwent fertility treatment of Ikaclomid, Pergonal, IVF, egg or sperm donation and/or Other but their miscarriage rates were nearly identical to the general population. Perhaps fertility treatment was not associated with higher rate of miscarriage because fertililty treatment often followed a relatively short history of not conceiving rather than a history of miscarriage.

Among the high risk Bedouin population of Beersheva Israel, Rh negative women experienced more stillbirths, even after receiving Anti-D [18] but miscarriage rates had not been analyzed. Our data describes a healthier population than that of Beersheva and found identical first trimester miscarriage rates for every blood type.

### Limitations

It is unknown whether the 18% empty fields for spontaneous miscarriage meant they had no miscarriages. This is hinted at by the fact that the overall miscarriage rate per pregnancy documented was 15%, the documented expected rate of miscarriage of recognized pregnancies, but cannot be known what their actual data was. The empty fields for miscarriage might be a byproduct of how busy the ward was or how quick the birth was.

Women who never reached 24 weeks of pregnancy were not included in this study. These women have been studied extensively elsewhere and were not relevant to the aim of this study, which was to get a first look at miscarriage rates per woman in a parous population. In most settings, one could expect a significant population of women who experience many miscarriages but no pregnancy of 24 weeks or more. However in Israel, the socialized medical program provides up to 3 IVF cycles per woman. In fact 4% of births in Israel are conceived using IVF. Therefore, the number of women who are interested in having a child, yet never achieve a 24 weeks pregnancy, is estimated to be minimal.

The study also does not differentiate between recurrent and non-recurrent miscarriages, as that was also not the aim of this study. The study also did not determine the effect of paternal age on miscarriage rates.

The strength of this study is the large size and tested reliability of the data. The population offers the opportunity to study a large population of parous women in a country with easily accessed, socialized medicine.

## Conclusions

43% of parous women experienced at least one spontaneous miscarriage. Miscarriage directly increases with age as well as parity. The more pregnancies, the more miscarriages are experienced. Grand multiparas have both more children and a higher prevalence of miscarriages. Those with 1–5 miscarriages also had an average of 3 living children, those with 6–16 miscarriages, had an average of 4 living children. Parous women until their late 30s, who have experienced multiple miscarriages, can be counseled that if they keep trying, they will likely carry a pregnancy to term.

## Abbreviations

CS: Cesarean surgery
